# 

**Published:** 1902-03-15

**Authors:** 


### ANNOUNCEMENTS.

### THE SOUTHWESTERN MICHIGAN DENTAL SOCIETY.

The Southwestern Michigan Dental Society will hold its spring meeting at Buchanan April 8th and 9th. A good program and a cordial reception is promised.

### GRAND RAPIDS (MICH.) DENTAL SOCIETY.

The Grand Rapids (Mich.) Dental Society held its annual meeting February 11, 1902, and elected the following officers: Pres., J. Ward House; V. P., H. D. DeWar; Sec'y, H. D. Watson. Ex. Com., L. F. Owen and W. A. Studley.

### OHIO VALLEY DENTAL SOCIETY.

A society with this name was re-organized at Wheeling, W. Va., January 23, 1902, and the following officers were elected; Pres., F. S. Maxwell; V. P., H. H. Harrison; Sec'y, J. H, McClure: Treas., J. G. Parr.

### MISSOURI STATE DENTAL ASSOCIATION.

The thirty-eighth annual meeting of the Missouri State Dental Association will be held at Jefferson City, May 21-23, 1902. Business meetings will be held in the legislative hall of the statehouse and the clinics will be held in the State penitentiary, thus insuring an abundance of clinical material. The papers to be read before the Association are of a most interesting character, and the meeting bids fair to be one of the best ever held in the State. It is certainly to be hoped that with the change in time of holding the meeting, and many other attractive features, the attendance will be all that can be desired. J. H. KENNERLY, *Sec'y*, St. Louis.

### ILLINOIS STATE DENTAL SOCIETY—COMMITTEE ON ART AND INVENTION.

The Committee on Art and Invention, of the Illinois State Dental Society, hereby invites and solicits a contribution of anything new in the way of appliances and inventions designed during the past year, which will be of interest to the profession in general. Everything submitted should be sent direct to the undersigned, with detailed description of its use and application, by April 1st, in order that it may receive proper classification and consideration in the presentation of the annual report at the coming meeting at Springfield, May 13-16th. The committee will care for and return each article submitted, but reserve the right to reject any which in their opinion may not be of practical value.

HART J. GOSLEE, *Chairman*,
580 Madison St., Chicago.

